# The Burden of Travel-Time and Distance Traveled for Hemodialysis Patients in Australian Major City Areas

**DOI:** 10.1016/j.ekir.2023.02.1077

**Published:** 2023-02-21

**Authors:** Stephen P. McDonald, Shahid Ullah, Kathryn Dansie, Emily Duncanson, Aarti Gulyani, Christopher E. Davies, Shilpanjali Jesudason

**Affiliations:** 1 Australia and New Zealand Dialysis and Transplant Registry, South Australia Health and Medical Research Institute, Adelaide, South Australia, Australia; 2 Faculty of Health and Medical Sciences, University of Adelaide, Adelaide, South Australia, Australia; 3 College of Medicine and Public Health, Flinders University, South Australia, Australia

## Introduction

The number of Australians receiving long-term dialysis for kidney failure is steadily increasing. Of the 13,051 patients undergoing dialysis at the end of 2017 in Australia, over 70% were receiving “facility” hemodialysis, at a hospital or satellite dialysis unit.^1^ In Australia, the type of dialysis facility is dependent on many complex factors, including patient clinical status and medical indications as well as dialysis capacity constraints and dialysis service structures. Typically, patients receive thrice weekly dialysis for 4 to 5 hours per treatment. In addition to time actually receiving hemodialysis, traveling to and from the treatment location is another physical, social, and financial burden for patients and their families, incurred at a high frequency typically for several years. Travel time and associated costs are a barrier to treatment adherence and access.^2^^,^^3^ Among those receiving hemodialysis, greater travel times have been associated with shortened and missed dialysis treatments, poorer quality of life, and increased mortality risk.^3^ Despite this, there are no published data on actual travel time or distance for patients receiving hemodialysis services in Australia and little elsewhere. For adults receiving facility-based hemodialysis treatment in a Major City in Australia (Supplementary Figure S1), we estimated travel distance and time from the population centroid of their residential postcode (postal area) to the treatment center (Supplementary Figure S2).

## Results

The characteristics of the cohort are shown in Supplementary Table S1, with no differences in age or comorbidity between hospital and satellite patients, apart from slightly longer kidney replacement therapy exposure in satellite patients. Overall, median 2-way travel distance to patients’ actual hemodialysis treatment facility was 18.3 km (interquartile range, 10.4–30.9 km), with substantial variation across locations (Table 1). Median estimated 2-way travel time was 32.6 minutes (interquartile range, 21.6–47.5 min). In contrast, the median 2-way travel distance and time to patients’ closest facility (from postal area population centroid) rather than actual treatment facility was 12.2 km (interquartile range, 7.2–19.4 km) and 23.8 min (interquartile range, 16.4–32.3 min), respectively. In all states and territories other than the Australian Capital Territory, median 2-way travel distance to patients’ actual facility was greater than that to their closest facility, with additional distances traveled ranging from 0 km (Australian Capital Territory) to 9.1 km (Queensland). Travel distances and times were also substantially greater for those patients (about 18% of the total cohort) who were in the initial 12 months of dialysis treatment compared with those receiving dialysis for over 12 months (21.1 vs. 17.8 km, *P* < 0.001; 36.4 vs. 31.8 min, *P* < 0.001).

**Table 1:** Patients 2-way travel distance and time to treatment facilities by Jurisdictions

Jurisdictions	*n*	Actual HD facility		Closest HD facility		Second closest HD facility		Third closest HD facility	
		Distance (km)	Time (min)	Distance (km)	Time (min)	Distance (km)	Time (min)	Distance (km)	Time (min)
ACT	146	11.3 (9.1–23.2)	20.0 (16.4–34.7)	11.3 (8.5–13.6)	19.3 (15.0–20.2)	25.5 (16.7–39.4)	37.9 (21.6–47.0)	31.2 (16.9–48.1)	39.9 (22.7–53.0)
NSW	1994	18.3 (9.8–31.4)	35.7 (22.3–52.1)	11.5 (7.0–18.9)	24.8 (17.1–34.2)	18.8 (13.3–31.4)	37.2 (28.1–46.4)	23.1 (17.3–42.8)	43.6 (35.3–61.3)
VIC	1787	16.4 (9.1–27.2)	29.7 (20.8–44.2)	9.3 (6.2–15.1)	20.7 (14.5–25.9)	15.7 (10.2–25.0)	30.0 (23.2–37.9)	21.5 (12.8–31.6)	35.6 (28.9–44.2)
QLD	945	24.5 (12.9–40.4)	37.4 (22.9–52.6)	15.2 (8.9–25.8)	27.2 (18.7–37.1)	25.1 (14.2–45.9)	38.4 (26.0–50.4)	31.7 (21.8–53.3)	46.0 (34.4–60.2)
SA	495	15.1 (10.1–24.0)	28.2 (19.0–40.5)	11.5 (7.1–16.0)	21.5 (15.2–28.9)	20.5 (13.6–28.6)	35.4 (28.5–43.0)	26.4 (21.5–37.8)	47.5 (38.6–56.4)
WA	675	21.1 (13.0–32.6)	31.4 (23.2–43.6)	13.7 (9.0–20.8)	23.7 (16.8–28.6)	25.1 (20.2–41.0)	35.8 (32.0–46.6)	34.6 (26.6–50.3)	46.8 (39.5–62.5)
Australia	6042	18.3 (10.4–30.9)	32.6 (21.6–47.5)	11.7 (7.0–18.7)	23.1 (16.2–31.3)	20.0 (12.9–32.0)	34.5 (26.5–45.2)	25.8 (17.3–40.8)	42.2 (33.6–54.6)
First yr of HD treatment	1101	21.1 (11.5–36.1)	36.4 (23.5–53.8)	11.8 (6.9–19.2)	23.1 (16.2–31.2)	20.3 (13.1–32.6)	34.7 (26.5–46.6)	25.8 (17.4–42.2)	42.1 (33.3–56.3)
Subsequent years of treatment	4941	17.8 (10.2–29.1)	31.8 (21.5–46.6)	11.7 (7.0–18.5)	23.1 (16.2–31.3)	19.8 (12.8–31.8)	34.4 (26.5–44.6)	25.9 (17.3–40.6)	42.2 (33.8–54.4)

ACT,Australian Capital Territory; HD, hemodialysis; km, kilometers; min, minutes; NSW, New South Wales; QLD, Queensland; SA, South Australia; VIC, Victoria; WA, Western Australia.

Values expressed as Median (25th to 75th percentile).

Sixteen percent of patients traveled more than 40 km as a round trip to their dialysis facility and a further 30% traveled between 20 and 40 km for each round trip (Table 2). Median estimated travel distance and time varied widely among facilities. Among all patients, less than half (44.2% by distance) were receiving treatment at their closest hemodialysis facility and about one-fifth (19.7% by distance) at their second closest facility. Among the 3371 patients not receiving treatment at their closest facility, about one-quarter (849) were receiving treatment in a hospital-based facility when the closest facility was a satellite facility and 782 were receiving treatment in a satellite facility when the closest facility was a hospital (35.6% of those receiving treatment in a satellite). Supplementary Table S2 shows the substantial differences in additional travel burden for patients not receiving treatment at their closest facility; for more than one-half of patients, this additional travel burden was over 10 km (Supplementary Table S3). Based on 3 hemodialysis treatments per week, receiving treatment at a facility other than the closest may result in 22 km or approximately 32 minutes of additional travel per week.

**Table 2:** Distance and time of travel to facility hemodialysis in major cities, by characteristics of treatment facility

Characteristic	Number (%)	
	Distance	Time
Patients traveling <10 km (<20 min) to treatment^a^	1443 (23.9%)	1324 (21.9%)
Patients traveling 10–20 km (20−40 min) to treatment^a^	1832 (30.3%)	2547 (42.2%)
Patients traveling 20–40 km (40−60 min) to treatment^a^	1798 (29.8%)	1327 (22.0%)
Patients traveling >40 km (>60 min) to treatment^a^	969 (16.0%)	844 (14.0%)
Treated at closest center	2671 (44.2%)	2639 (43.7%)
Treated at second closest center	1192 (19.7%)	1127 (18.7%)
Treated at third closest center	668 (11.1%)	684 (11.3%)
Patients not treated at closest center	3371 (55.8%)	3403 (56.3%)
Receiving treatment at hospital	1177 (34.9%)	1230 (36.1%)
With closest being a satellite unit	849 (72.1%)	953 (77.5%)
Receiving treatment at satellite center	2194 (65.1%)	2173 (63.9%)
With closest being a hospital	782 (35.6%)	618 (28.4%)

Kms, kilometers.

Values expressed according to distance (kilometers) and time (minutes).

a The figures for Time column represents the time cut of point in the parentheses.

## Discussion

Hemodialysis imposes a substantial time burden on patients’ quality of life; the median prescribed dialysis session in Australia is 4.5 hours, thrice weekly.^4^ In this study, we documented an additional element of this burden: travel time to and from treatment. On average, adult facility hemodialysis patients in Australian major cities spend 1.5 hours per week traveling, in addition to the actual time spent receiving treatment. In addition, we demonstrated a higher travel burden for patients in their first year of treatment and substantial variation (within major cities) in travel burden between actual and next closest facilities and between states.

Minimization of travel time is an important goal for patients but given the sizable group of patients who travel beyond their closest facility to receive dialysis, that goal has clearly not been met. Approximately one-third of these patients travel over 20 km extra to dialyze; in terms of time, approximately one-quarter travel an additional 20 to 39.9 minutes and 17% travel an additional 40 minutes or more. This mismatch between the distribution of facilities and demand suggests an opportunity to alleviate patient burden and improve quality of life.

For many patients not being treated at their closest facility, there is a discrepancy between the type of facility attended and those closest to their residence. There are likely to be 2 main components to the group who travel extra distance to dialyze at a hospital-based facility: patients who require access to the higher level of care available in a hospital-based facility and those who have recently commenced chronic hemodialysis treatment and have not yet been allocated a place at a satellite facility. The former phenomenon was broadly assessed by analyzing comorbidities and age of patients in satellite and hospital facilities, with no differences in case-mix observed. The latter phenomenon reflects congestion and limitations in available dialysis places; these are factors currently common to all Australian jurisdictions. For some patients, there may be other explanations in some cases such as personal preferences and employment-related issues. However, the Australia and New Zealand Dialysis and Transplant registry does not collect data on the reasons for patients dialyzing in a particular facility or whether patients are on a waiting list for another facility.

Transportation and the perceived need to live close to a dialysis unit have been identified by patients as significant logistical and psychosocial stressors when approaching and preparing for initiation of hemodialysis.^5^ In a 2011 Australian survey of 1505 patients who dialyzed at a hospital or satellite unit, 69% traveled by car, either driven by themselves or a family member.^6^ The majority of patient-reported spending on transport ranged from $10 to $50 AUD per week, and reduced travel and avoiding family burden of travel to dialysis were among the most commonly reported reasons for patients choosing home-based peritoneal dialysis over hemodialysis.

This study has limitations, particularly the use of postcode as a proxy for residential address. We used estimates based on postal area population centroids to calculate travel distance and time. Although the overall average distance will be accurate, there may be misclassification of some individuals at the outer edges of a postcode who are in fact traveling to their closest dialysis unit. We used estimates at the typical travel times for facility hemodialysis patients but the actual start and stop times of dialysis are not collected by the registry. Estimates also assumed travel by car. Modes of transport to access dialysis are likely to be varied, including bus, train, and ambulance^6^ but these other methods are likely to be more time consuming. Finally, estimates of travel time and distance were obtained using Google Maps and are calculated using a combination of geospatial data and actual travel time from mobile phone users through a proprietary process.^7^

Our findings raise areas for future research. Our analysis did not capture factors that may contribute to longer travel times or hospital-based treatment despite a closer satellite facility, such as the level of clinical care needs during treatment, availability and structure of hemodialysis services, dialysis capacity constraints and staffing, patient preferences, or mode of transport. These are all important factors that remain to be explored but undoubtedly influence the findings we report in this analysis. The effects of distance on health outcomes, including choice of facility over home hemodialysis, wait listing, and access to transplantation remain uncertain and this data should be captured potentially in registry datasets. This study was restricted to major cities because of the limitations of postal area analysis in large rural areas; however, the enormous burden of travel and even relocation to access dialysis care experienced by patients located in rural areas is important to define in future work. We could not identify similar studies internationally to examine whether the situation we describe is better or worse than that in other countries.

In summary, through application of geospatial analysis to the Australian hemodialysis population, this study provides insight into a previously suspected but unmeasured aspect of the dialysis patient experience. Travel burden and equitable access to dialysis should be major priorities when developing new dialysis infrastructure and future research should explore patient-, service-, and state-level factors that influence whether a patient can have dialysis closest to home.

## Disclosure

All the authors declared no competing interests.

## Supplementary Material

**Supplementary Methods.**

**Supplementary Reference.**

**Figure S1.** Flow diagram of inclusion criteria.

**Figure S2.** Illustration of population centroid (as opposed to geographic centroid) of postal area.

**Table S1.** Characteristics of patients included in the analyses.

**Table S2.** Two-way additional travel distance and time between actual and nearest dialysis facilities for patients not receiving treatment at their closest center.

**Table S3.** Additional travel distance and time for patients not receiving treatment at their closest facility.
