# Supplementary material for: The Burden of Travel-Time and Distance Traveled for Hemodialysis Patients in Australian Major City Areas
Source: Kidney Int Rep. 2023 Feb 21;8(5):1105–8. doi: 10.1016/j.ekir.2023.02.1077 (PMC10166733; doi:10.1016/j.ekir.2023.02.1077)

Online material

Supplement: Methods

The Australia and New Zealand Dialysis and Transplant (ANZDATA) Registry is a Clinical Quality Registry that collects information about people with KF treated with dialysis or kidney transplantation. All renal units in Australia and New Zealand participate. A deidentified data extract from ANZDATA was used to examine a cohort of Australian haemodialysis patients. The study cohort consisted of patients treated at adult facilities with haemodialysis in major cities of Australia at 31 December 2017 (Supplementary Figure S1). Facilities based in paediatric renal units were excluded from this study - the specialised nature of haemodialysis in this group means travel times and distances will be determined by availability of highly specialised paediatric tertiary hospitals rather than community based haemodialysis facilities. 'Major cities' were defined based on postcode using the Australian Bureau of Statistics Remoteness classification (S1). Outliers with unfeasibly long distances or travel to non-adjacent states were excluded (e.g. patients travelling long distance due to temporary relocations). The 'actual facility' was defined as the facility at which the patient was receiving haemodialysis at 31 December 2017. The data extract included demographic information (including postcode of residence), comorbidities and treatment facility type (hospital vs satellite). In Australia, satellite haemodialysis facilities are those with lower staffing levels intended to provide care closer to home for chronic, lower-acuity patients. Locations with both hospital and satellite haemodialysis facilities on the same physical site (such as multiple on the same hospital campus) were classified as being hospital haemodialysis facilities. The cohort was categorised into those who were less than 12 months since the start of dialysis, and the remainder greater than 12 months.

As ANZDATA collects residential postcode but not actual residential addresses, 2016 Census population weighted centroids were calculated for each postal area (POA) corresponding to the postcode. POAs are an approximation of Australia Post postcodes produced by the Australian Bureau of Statistics; the boundaries produced for POAs are constructed from Statistical Area Level 1 (S2) areas. We calculated the population weighted centroid for each postcode based on spatial data (S3). The use of population weighted (rather than geographic) centroids for calculation of travel distances assumes all people were living at the population centroid of their POAs, and accounts for the differing population distribution within a POA rather than assuming all people live at the geographic centre (Figure 2). This study was limited to POAs in Major Cities. Use of POAs for people in rural and remote areas poses a number of challenges including small residential numbers, the large area and possible errors associated with relocation closer to centres of treatment.

The two-way distance and travel time (by car) from the population weighted centroid of residential postcode to treatment facility was estimated utilising Google Maps Directions API (S4) for four different time points on a weekday – 7:00 am to facility; 12:00 pm to Home; 12:30 pm to facility and 5:30 pm to Home. These times were chosen to approximate the times and directions for travel to morning and afternoon dialysis shifts. We restricted this analysis to "major cities" as POA Google Maps use a proprietary algorithm to calculate the shortest distance and time between 2 points; the data for the latter is based on actual travel patterns.

All statistical analyses were conducted using R version 3.4.3 (S5) and Stata version 15.1 (College Station, TX). For each person this was calculated for the actual treatment facility, and also for the three closest haemodialysis facilities, by distance and also by time. The candidate haemodialysis facilities for being the first, second or third closest included all adult facilities in major cities of Australia with patients at 31 December 2017. Travel times and distances were expressed as medians (interquartile range (IQR)). Mann-Whitney U tests were conducted to test the difference of outcomes between groups given the skewed nature of the data.

Ethics approval for ANZDATA operations is from the Central Adelaide Local Health Network Ethics Committee (HREC Reference number HREC/17/RAH/408, CALHN Reference number R20170927).

Supplementary References

S1. Australian Bureau of Statistics. Australian Statistical Geography Standard (ASGS): Volume 5 - Remoteness Structure, July 2016. Canberra: Australian Bureau of Statistics; 2018.

S2. Australian Bureau of Statistics. Australian Statistical Geography Standard (ASGS). Cat 1270.0.55.003. Canberra: Australian Bureau of Statistics; 2011.

S3. Hanigan I, Hall G, Dear KB. A comparison of methods for calculating population exposure estimates of daily weather for health research. Int J Hlth Geographics. 2006;5(1):38.

S4. Google Developers. Distance Matrix API Overview: Google, Mountain View, CA; [Available from: https://developers.google.com/maps/documentation/distance-matrix/overview.

S5. R Core Team. R: A language and environment for statistical computing. . Vienna: R Foundation for Statistical Computing, ; 2016.

Supplementary Table S1. Characteristics of patients included in the analyses.

(A) Overall Cohort

**N**	6042
**Age [Median, IQR]**	69 [57-77]
**Gender (female)**	37.5%
**Location of dialysis**	28.4% hospital, 71.6% satellite facility
**Within 12 months of commencement of dialysis**	18.2%
**Comorbidities:**	
Coronary artery disease	50.0%
Chronic lung disease	20.3%
Cerebrovascular disease	19.4%
Peripheral Vascular Disease	33.2%
Diabetes	51.4%
**Primary kidney disease**	
Diabetic kidney disease	35.8%
Glomerular disease	21.2%

(B) According to type of facility

		Hospital	Satellite	p-value
N		1718	4324	
Diabetes	no	864 (50.3%)	2071 (47.9%)	0.080
	yes	849 (49.4%)	2249 (52.0%)	
		5 (0.3%)	4 (0.1%)	
Coronary disease	no	857 (49.9%)	2160 (50.0%)	0.99

	yes	857 (49.9%)	2159 (49.9%)	
		4 (0.2%)	5 (0.1%)	
Cerebrovascular disease	no	1365 (79.5%)	3492 (80.8%)	0.33
	yes	346 (20.1%)	826 (19.1%)	
		7 (0.4%)	6 (0.1%)	
Peripheral vascular disease	no	1110 (64.6%)	2915 (67.4%)	0.052
	yes	600 (34.9%)	1402 (32.4%)	
		8 (0.5%)	7 (0.2%)	
Years receiving KRT, median (IQR)		3.0 (0.8, 6.8)	3.8 (1.7, 7.5)	<0.001
Age, median (IQR)		69.0 (56.0, 78.0)	69.0 (58.0, 77.0)	0.33

Supplementary Table S2 . Two-way additional travel distance and time between actual and nearest dialysis facilities for patients not receiving treatment at their closest centre

Characteristic	Median additional travel between actual and closest facility			
	N	Distance	N	Time
**ACT**	56	4.3 (0.9-14.3)	50	7.5 (4.9-14.9)
**NSW**	1074	11.1 (3.8-21.4)	1101	17.5 (6.3-33.9)
**VIC**	1102	10.8 (3.7-23.5)	1111	16.0 (5.1-32.6)
**QLD**	587	9.1 (1.1-22.1)	585	12.8 (3.0-28.0)
**SA**	232	9.0 (5.8-17.7)	231	15.6 (9.4-24.6)
**WA**	320	16.4 (8.7-32.7)	325	20.2 (10.9-31.1)
**Australia**	3371	10.7 (3.8-22.7)	3403	15.9 (5.9-31.0)
**First year of treatment**	702	14.0 (5.6-28.2)	717	21.0 (8.4-35.9)
**Subsequent years of treatment**	2669	9.9 (3.4-21.6)	2686	15.0 (5.7-29.2)

Values expressed as Median (25th and 75th percentile) according to distance (kilometres) and time (minutes).

Supplementary Table S3. Additional travel distance and time for patients not receiving treatment at their closest facility

Travel	Number (%)
**Distance**	**3371 (55.8%)**
<5 km	989 (29.3%)
5-9.9 km	623 (18.5%)
10-19.9 km	763 (22.6%)
≥20 km	996 (29.5%)
**Time**	**3403 (56.3%)**
<20 min	1968 (57.8%)
20-39.9 min	867 (25.5%)
≥40 min	568 (16.7%)

Supplementary Figure S1. Flow diagram of inclusion criteria

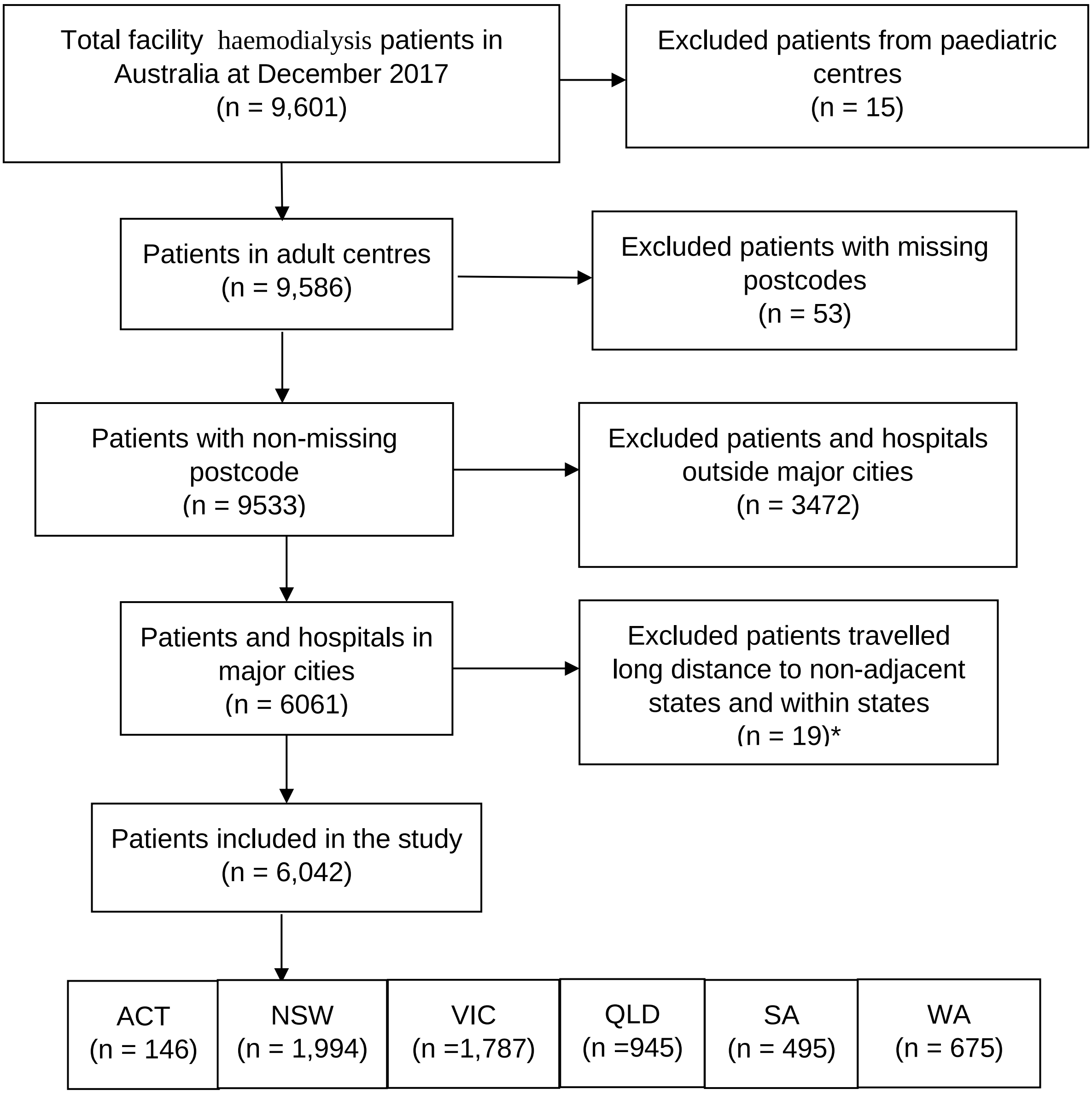

Supplementary Figure S2 Illustration of population centroid (as opposed to geographic centroid) of postal area. All travel times and distances were calculated from the population centroid to the closest dialysis facility (A) and the actual facility utilised (B).

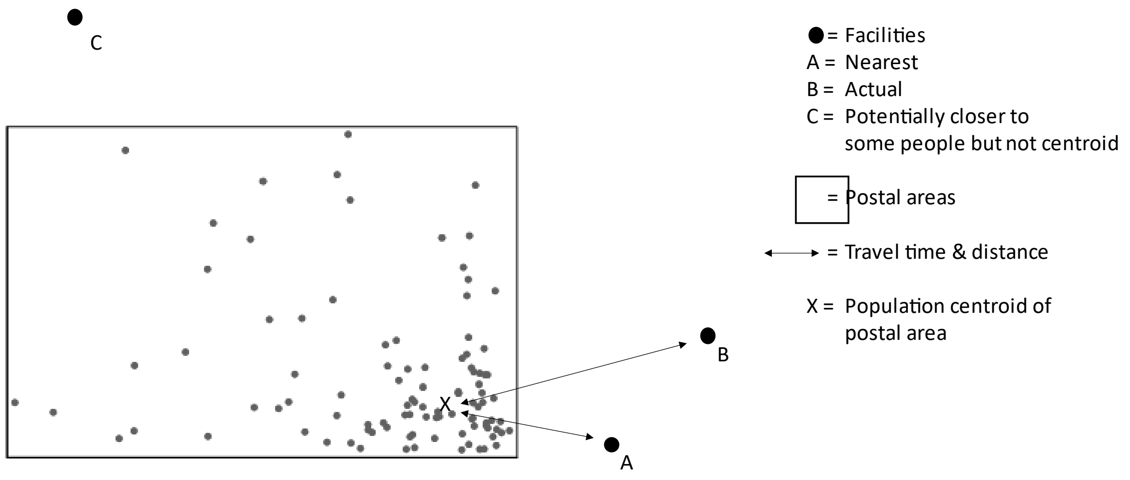

Supplement: Supplementary File (PDF) [file mmc1.pdf]
